# Retention in care among older adults living with HIV in western Kenya: A retrospective observational cohort study

**DOI:** 10.1371/journal.pone.0194047

**Published:** 2018-03-28

**Authors:** Jepchirchir Kiplagat, Ann Mwangi, Alfred Keter, Paula Braitstein, Edwin Sang, Joel Negin, Charles Chasela

**Affiliations:** 1 School of Medicine, College of Health Sciences, Moi University, Eldoret, Kenya; 2 Academic Model Providing Access to Healthcare (AMPATH), Eldoret, Kenya; 3 Department of Epidemiology and Biostatistics, School of Public Health, Faculty of Health Sciences, University of Witwatersrand, Johannesburg, South Africa; 4 Dalla Lana School of Public Health, University of Toronto, Toronto, Canada; 5 School of Public Health, University of Sydney, Sydney, Australia; 6 Right to Care, EQUIP, Centurion, Pretoria, South Africa; The Ohio State University, UNITED STATES

## Abstract

**Background:**

Retention, defined as continuous engagement in care, is an important indicator for quality of healthcare services. To achieve UNAIDS 90-90-90 targets, emphasis on retention as a predictor of viral suppression in patients initiated on ART is vital. Using routinely collected clinical data, the authors sought to determine the effect of age on retention post ART initiation.

**Methods:**

De-identified electronic data for 32965 HIV-infected persons aged ≥15 years at enrolment into the Academic Model Providing Access to Healthcare program between January 2008 and December 2014 were analyzed. Follow-up time was defined from the date of ART initiation until either loss to follow-up or death or close of the database (September 2016) was observed. Proportions were compared using Pearson’s Chi-square test and medians using Mann-Whitney U test. Logistic regression model was used to assess differences in ART initiation between groups, adjusting for baseline characteristics. Cox proportional hazards model adjusting for baseline characteristics and antiretroviral therapy (ART) status was used to compute hazard ratios. Kaplan-Meier survival function was used to compare retention on ART at 12, 24, and 36 months post ART initiation.

**Results:**

Of the total sample, 3924 (12.0%) were aged ≥50 years at enrolment. The median (IQR) age of young adults and older adults were 32.5 (26.6, 36.9) and 54.9 (51.7, 59.9) respectively. ART initiation rates were 70.5% among older adults and 68.2% among younger adults. Retention rates in care at 12, 24 and 36 months post ART initiation were 73.9% (95% CL: 72.2, 75.5), 62.9% (95% CL: 61.0, 64.7) and 55.4% (95% CL: 53.5, 57.3) among older adults compared to 69.8% (95% CL: 69.1, 70.4), 58.1% (95% CL: 57.4, 58.8) and 49.3% (95% CL: 48.6, 50.0) among younger adults (p <0.001). A higher proportion of older adults were retained in HIV care post ART initiation compared to younger adults, Adjusted Hazard Ratio (AHR): 0.83 (95% CI: 0.78, 0.87) though they were more likely to die, AHR: 1.35 (95% CI: 1.19, 1.52).

**Conclusion:**

A higher proportion of older adults are initiated on ART and have better retention in care at 12, 24 and 36 months post ART initiation than younger adults. However, older adults have a higher all-cause mortality rate, perhaps partially driven by late presentation to care. Enhanced outreach and care to this group is imperative to improve their outcomes.

## Introduction

Globally, there are more than 36.7 million people living with HIV [1] and over half of these on anti-retroviral treatment (ART). Recent data from WHO and UNAIDS [2, 3] indicate a reduction in new HIV infections and a concurrent increase in the numbers initiating ART. The widespread initiation of ART has significantly increased the life expectancy of people living with HIV. Consequently, the number of older HIV-infected adults have progressively increased in the last decade [4]. This growth has been attributed to longer survival among HIV-infected and new infections occurring among those aged 50 years and above [5]. Despite the increasing numbers in Sub-Saharan Africa (SSA), less attention is given to older adults who are HIV infected, with lack of adequate data on their engagement in the HIV care continuum. As a result, there remains a significant evidence and policy gap in the global HIV response [6].

Finding HIV-infected persons, initiating them on treatment, and providing long term care is critical in the fight against the HIV epidemic. UNAIDS in its target to end the HIV epidemic by 2030 has focused on the 90-90-90 strategy: diagnosing 90% of those infected with HIV, initiation 90% of the diagnosed on ART and achieving viral suppression in 90% of those on ART [7]. Albeit ambitious, these targets are achievable, and require continuous engagement in care for all diagnosed with HIV and initiated on treatment. Persons living with HIV can, however, be lost at any stage in the care continuum [8], presenting challenges to retention in HIV care programs. Retention, defined as active engagement in care, is therefore vital in reducing morbidity and mortality, preventing new infections and achieving viral suppression [9, 10]. It is also an indicator of quality service provision.

Data on retention in care among HIV infected persons after ART initiation [11–13] and at pre-ART phase of care [11, 14–18] has been well documented. However, there is limited information comparing retention among younger and older adults. Though ART guidelines have evolved over time, there are challenges when managing HIV-infected older adults. Prior to initiating ART, possible toxicities and adverse events are carefully examined as older adults have a higher risk of comorbidities [19] than the younger age group. These factors are likely to increase morbidity and in turn affect retention among older adults after starting ART. Using routinely collected clinical data, we determined the effect of age on retention in care and all-cause mortality.

## Material and methods

### Study setting

This study is based on a cohort of HIV infected patients enrolled in the Academic Model Providing Access to Healthcare (AMPATH) program. The AMPATH Program was initiated in 2001, in Eldoret Kenya, as a joint partnership between Moi University School of Medicine, Indiana University School of Medicine, and the Moi Teaching and Referral Hospital. In 2004, the program received funding from USAID that resulted in the ‘USAID-AMPATH Partnership’. AMPATH’s goals at the time of its initiation was to establish an HIV care system that would serve the needs of patients in western Kenya and to assess the barriers to, and outcomes of ART. Einterz and team have described the program in detail [20]. In brief, the program has established an HIV care system in western Kenya that has enrolled over 160,000 persons living with HIV with about 90% of them on ART. It operates in 135 Ministry of Health (MOH) facilities spread throughout the western part of the country. AMPATH is currently the largest program providing free HIV care and treatment in the country [21].

### Study design, participant inclusion and exclusion criteria

This study was a retrospective cohort design that used records on patients enrolled for HIV care at all AMPATH facilities between January 1, 2008 and December 31, 2014. Patients with confirmed HIV infection, aged ≥15 years with at least one follow-up visit were included. Transfers into the facilities were excluded. Patients were categorized into younger adults (<50 years) and older adults (≥ 50 years).

### Data sources

The data was obtained from the AMPATH Medical Records System (AMRS). The AMPATH clinical care protocols for managing HIV infection are consistent with WHO recommendations and have been described in detail elsewhere [22, 23]. Standardized clinical encounter forms are completed by healthcare providers and collect information on patients’ demographic, clinical, and treatment information at each encounter. The data assistants enter the data from the clinical encounter forms into the AMRS, a secure computerized database designed for clinical management [24, 25]. To validate the entered data, a random sample of 10% of the forms entered is reviewed. At the time of registration, patients are provided with a unique identifying number, and prior to analysis, all data were stripped of identifying information.

Use of de-identified patient-level data obtained from AMPATH was approved by the ethics review committee (ERC) of the University of Witwatersrand (Clearance Certificate No: *M160449*), the Moi University and Moi Teaching and Referral Hospital Institutional Research Ethics Committee–IREC (Formal Approval No. *FAN*: *IREC 1664*) and the AMPATH administration. Patient consent was waived by both ERCs.

### Variables

At enrollment to the HIV care program we obtained information on; date of birth, gender, weight, height, enrolment date, marital status, year of HIV diagnosis, educational level, point of HIV testing, disclosure status, travel time to the clinic, medication at enrollment, CD4 cell count, viral load, WHO staging and opportunistic infections, and co-morbidities. For those who were initiated on ART we obtained information on: date of ART initiation, gender, body mass index (BMI), time to ART initiation (calculated from enrolment date), date of ART regimen switching or discontinuation, reasons for ART switch or discontinuation. Other repeated measurements and clinical events including: WHO clinical staging, CD4 cell counts, BMI measurements, use of prophylaxis, number of hospitalizations, clinic and ART adherence, incidence of HIV and non-HIV related co-morbidities were also obtained. Our primary outcome measure was retention in care at 12, 24 and 36 months post ART initiation. LTFU was defined as patients who are not recorded as dead or transferred out but have not been seen in care in 3 months or more after their last date of appointment for those on ART.

CD4 measurement collected within 90 days of the date of enrollment was considered as the baseline value. CD4 measured within plus or minus 90 days from the date of ART initiation was considered as the CD4 measurement at ART start. Body Mass Index (BMI) and WHO clinical stage were extracted within 30 days from the date of enrollment and within 30 days post-ART start and 90 days prior to ART start to give us the covariates at baseline and at ART start respectively. Advanced stage of HIV was defined as presenting with CD4 <200 cells per mm^3^ or WHO stage III/IV.

### Statistical analysis

Data management and statistical analysis were done using STATA version 13 SE (College Station, Texas 77845 USA). We examined each variable for completeness and differences in proportions of missing data were compared between the younger (<50 years) and the older (≥ 50) adults. We used descriptive statistics such as frequencies and the corresponding percentages to summarize categorical variables. Descriptive statistics which include median and interquartile range (IQR) were used to summarize continuous variables due to violation of Gaussian assumptions. The Gaussian assumptions were assessed using Shapiro-Wilks test.

Association between the categorical variables was assessed using Pearson’s Chi-Square test. Association between binary variables and continuous variables was assessed using Mann-Whitney U test. Kaplan-Meier survival distribution curves were used to describe and compare the survival rates between the older adults and the younger adults. Log-rank test was used to compare the survival functions.

Factors associated with retention and death were assessed using Cox proportional hazards regression model. Hazard ratios (HR) and the corresponding 95% confidence limits (95% CL) were reported. Logistic regression model was used to assess factors associated with ART initiation. We report the odds ratios (OR) and the corresponding 95% CL. All tests of significance with p-value < 0.05 were considered statistically significant.

We used inverse probability weighting (IPW) to correct for potential biases introduced due to missing covariates data [26]. The key clinical covariates, CD4, WHO clinical stage and BMI had missing covariate data. Hence we used the baseline variables that were fully observed (age, gender, and clinic location), composite outcome of loss to follow up or death, and follow-up time for all the participants to develop a propensity score model to use for weighting the outcome models. Up to 49.2% of the participants had missing covariate data for either of CD4, WHO clinical stage or BMI. We calculated the weights using binary logistic regression model and performed the necessary diagnostics to ensure that there were no extreme weights. The minimum and maximum weights were 0.7 and 1.5 respectively. Our assumption was that the missing data are missing at random (MAR) and that the probability of missing information can be fully explained by the covariates that are fully observed and by the information on mortality and loss to follow-up.

## Results

### Characteristics of the study population

There were 32,965 HIV-infected persons aged 15 years and above at enrolment who were eligible for analysis, 3942 (12.0%) were aged ≥ 50 years. Compared to young adults, a significantly higher proportion of the older adults were male (47.5% vs. 32.1%, p<0.001) attended rural clinics (61.1% vs. 54.7%, p<0.001), had lower CD4 count levels (p<0.001), were underweight (p<0.001) and were more likely to be enrolled in care late into their HIV infection (WHO clinical stage III/IV, p<0.001). A lower proportion of the older adults had disclosed their HIV status at the time they enrolled in the AMPATH clinics (63.6% vs. 70.4%, p<0.001), and a higher proportion were receiving prophylaxis for treatment of opportunistic infections (57.4% vs. 54.2%, p<0.001). Older adults were more likely to have been tested for HIV at the hospital or healthcare facility (p<0.001) as opposed to testing at VCT or mVCT. Patient characteristics at enrollment are presented in “Table 1”.

**Table 1:** Patient characteristics at enrolment stratified by age <50 and ≥50 years.

		<50 years (n = 29023, 88.0%)	≥50 years (n = 3942, 12.0%)	
Variable	n	Median (IQR) or n (%)	Median (IQR) or n (%)	P–value
Age (years)	32965	32.5 (26.6, 38.9)	54.9 (51.7, 59.9)	NA
Male	32965	9328 (32.1%)	1874 (47.5%)	<0.001
Married	32556	14420 (50.3%)	1879 (48.1%)	0.009
Years of school	27900	8.0 (7.0, 10.0)	7.0 (5.0, 10.0)	<0.001
Urban clinic location	32965	13139 (45.3%)	1534 (38.9%)	<0.001
CD4 cells per mm^3^	24494	268.0 (107.0, 470.0)	219.0 (93.0, 385.0)	<0.001
<200		8535 (39.7%)	1384 (46.6%)	
200–499	24494	8189 (38.1%)	1159 (39.0%)	<0.001
≥500		4797 (22.3%)	430 (14.5%)	
BMI (kg/m^2^)	23102	20.0 (18.0, 22.3)	19.4 (17.4, 21.7)	<0.001
<18.5		6273 (30.8%)	1088 (39.5%)	
18.5–25.0	23102	12069 (59.3%)	1407 (51.1%)	<0.001
25.0–30.0		1663 (8.2%)	195 (7.1%)	
>30.0		341 (1.7%)	66 (2.4%)	
WHO clinical stage				
Stage I& II	29124	17193 (67.3%)	2111 (59.4%)	<0.001
Stage III& IV		8376 (32.7%)	1444 (40.8%)	
Disclosed HIV status	32016	19851 (70.4%)	2431 (63.6%)	<0.001
On TB treatment	30822	2002 (7.4%)	278 (7.5%)	0.777
Receiving OI prophylaxis	30824	14708 (54.2%)	2123 (57.4%)	<0.001
Point of testing				
pMTCT	21280	2292 (12.2%)	20 (0.8%)	
VCT/mVCT,		7025 (37.3%)	924 (38.1%)	<0.001
Hospital/healthcare facility		9538 (50.6%)	1481 (61.1%)	

pMTCT–prevention of Mother-to-Child Transmission.

VCT–Voluntary Counselling and Testing.

mVCT–mobile Voluntary Counselling and Testing.

OI–Opportunistic Infections.

### ART initiation

At the time of ART initiation, majority of older adults were male (47.0% vs. 29.9%, p<0.001), were attending the rural clinics (61.9% vs. 55.5%, p<0.001), and had lower CD4 cell count, median: 168.0 (IQR: 80.0, 268.0) vs. 190.0 (IQR: 81.0, 323.0) cells per mm^3^. The WHO clinical stage was worse (WHO stage III and IV) for the older adults (p<0.001). Detailed characteristics of participants at ART initiation are summarized in “Table 2”.

**Table 2:** Patient characteristics at ART initiation among those who initiated ART stratified by age <50 and ≥50 years at enrolment.

		<50 years (n = 19799)	≥50 years (n = 2783)	
Variable	n	Median (IQR) or n (%)	Median (IQR) or n (%)	P–value
Age at ART start (Years)	22582	33.6 (27.6, 39.9)	55.5 (52.3, 60.4)	
Male	22582	5928 (29.9%)	1307 (47.0%)	<0.001
Married	22454	10341 (52.5%)	1360 (49.1%)	0.001
Years of school	19219	8.0 (7.0, 11.0)	7.0 (5.0, 11.0)	<0.001
Urban clinic location	22582	8803 (44.5%)	1061 (38.1%)	<0.001
CD4 cells per mm^3^	15749	190.0 (81.0, 323.0)	168.0(80.0, 268.0)	<0.001
<200		7206 (52.4%)	1178 (59.2%)	
200–499		5083 (36.9%)	749 (37.7%)	<0.001
≥500		1471 (10.7%)	62 (3.1%)	
BMI at ART start (kg/m^2^)	13016	20.1 (17.9, 22.5)	19.2 (17.4, 21.5)	<0.001
<18.5		3597 (31.2%)	607 (40.4%)	
18.5–25.0		6664 (57.9%)	767 (51.0%)	<0.001
>25.0		1035 (1.9%)	94 (2.3%)	
WHO clinical stage				
Stage I&II	22120	11903 (61.5%)	1482(53.6%)	<0.001
Stage III&IV		7452 (38.5%)	1283 (46.4%)	

**Fig 1:**
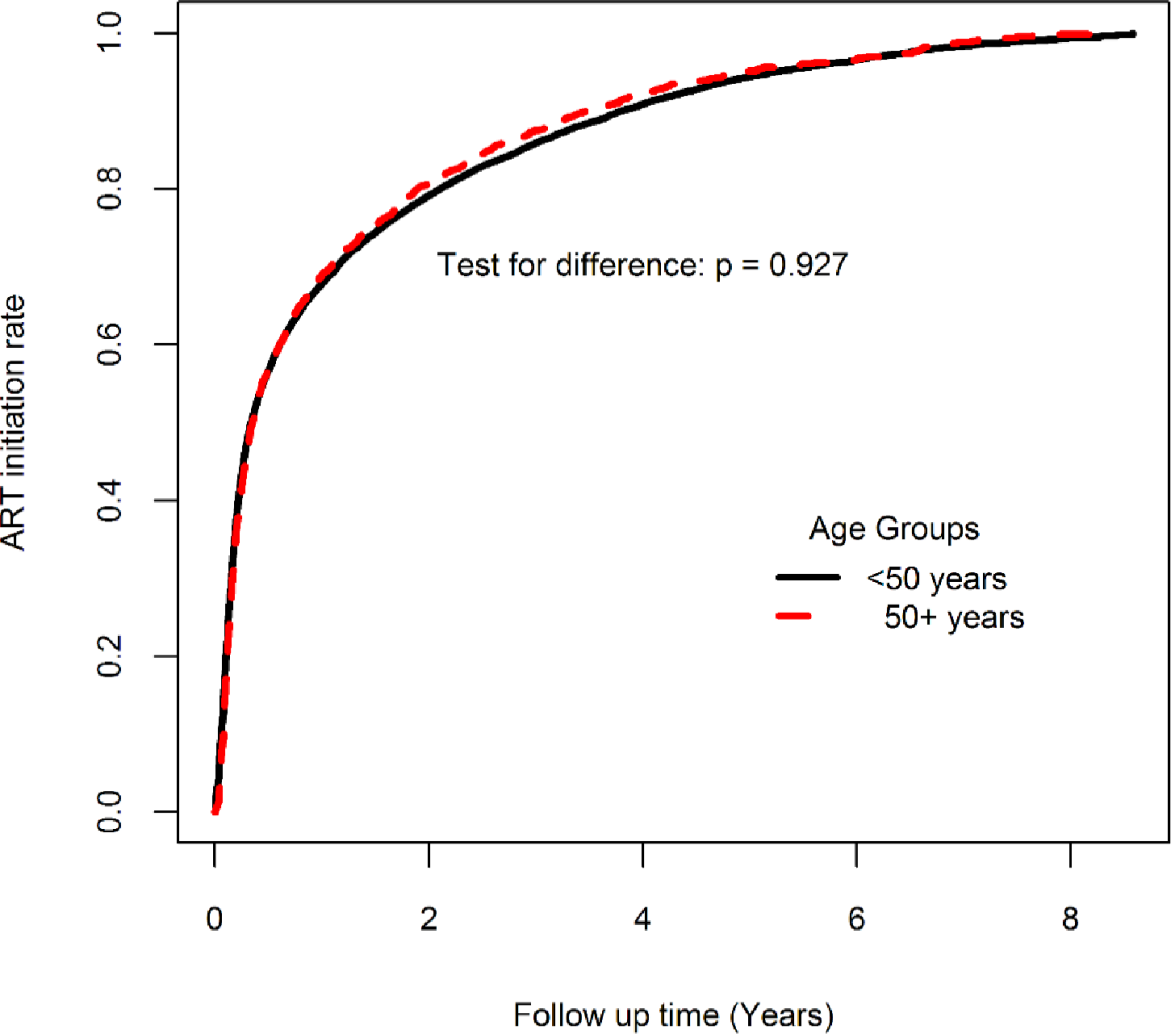
ART initiation rates by age group.

During follow-up, the rate of new ART initiation at any time during the follow-up was similar for younger and older adults, p = 0.927 “Fig 1”. However, the proportion of the older adults who were on ART at the close of the study was significantly higher (70.6% vs. 68.2%, p<0.001).

A significantly higher proportion of older adults (70.5%) were started on ART, compared to younger adults (68.2%). Male participants and participants attending the urban clinic at enrollment into care were 34% and 10% less likely to be started on ART respectively. In “Table 3”, participants who were married, on opportunistic infection (OI) prophylaxis and enrolled at an advanced stage of HIV infection had increased odds of being initiated on ART.

**Table 3:** Factors associated with ART initiation.

^†^Variable	n	Unadjusted OR (95% CL)	^ħ^Adjusted OR (95% CL)
Aged ≥50 vs. aged <50 years	32965	1.12 (1.04, 1.20)	1.13 (1.03, 1.25)
Male	32965	0.76 (0.73, 0.80)	0.66 (0.61, 0.70)
Urban clinic location	32965	0.90 (0.86, 0.94)	0.90 (0.88, 0.99)
Married	32556	1.30 (1.24, 1.37))	1.54 (1.45, 1.65)
Using OI prophylaxis at baseline	30824	1.25 (1.20, 1.32)	1.30 (1.22, 1.38)
Advanced HIV status	31634	1.86 (1.77, 1.96)	2.53 (2.36, 2.70)
Hospitalized prior to ART start	28320	0.75 (0.61, 0.92)	0.66 (0.52, 0.83)
Disclosed HIV status at baseline	32016	1.04 (0.99, 1.10)	0.93 (0.87, 1.00)
Sample size			25447

^†^Variables measured at enrollment.

Advance HIV status = CD4 < 200 cells / mm^3^ or WHO clinical stage 3 or 4.

^ħ^Weighted for missing data on CD4 count, WHO clinical stage and BMI

OR–Odds Ratio, 95% CL: 95% Confidence Limits.

### Retention post ART initiation

**Fig 2:**
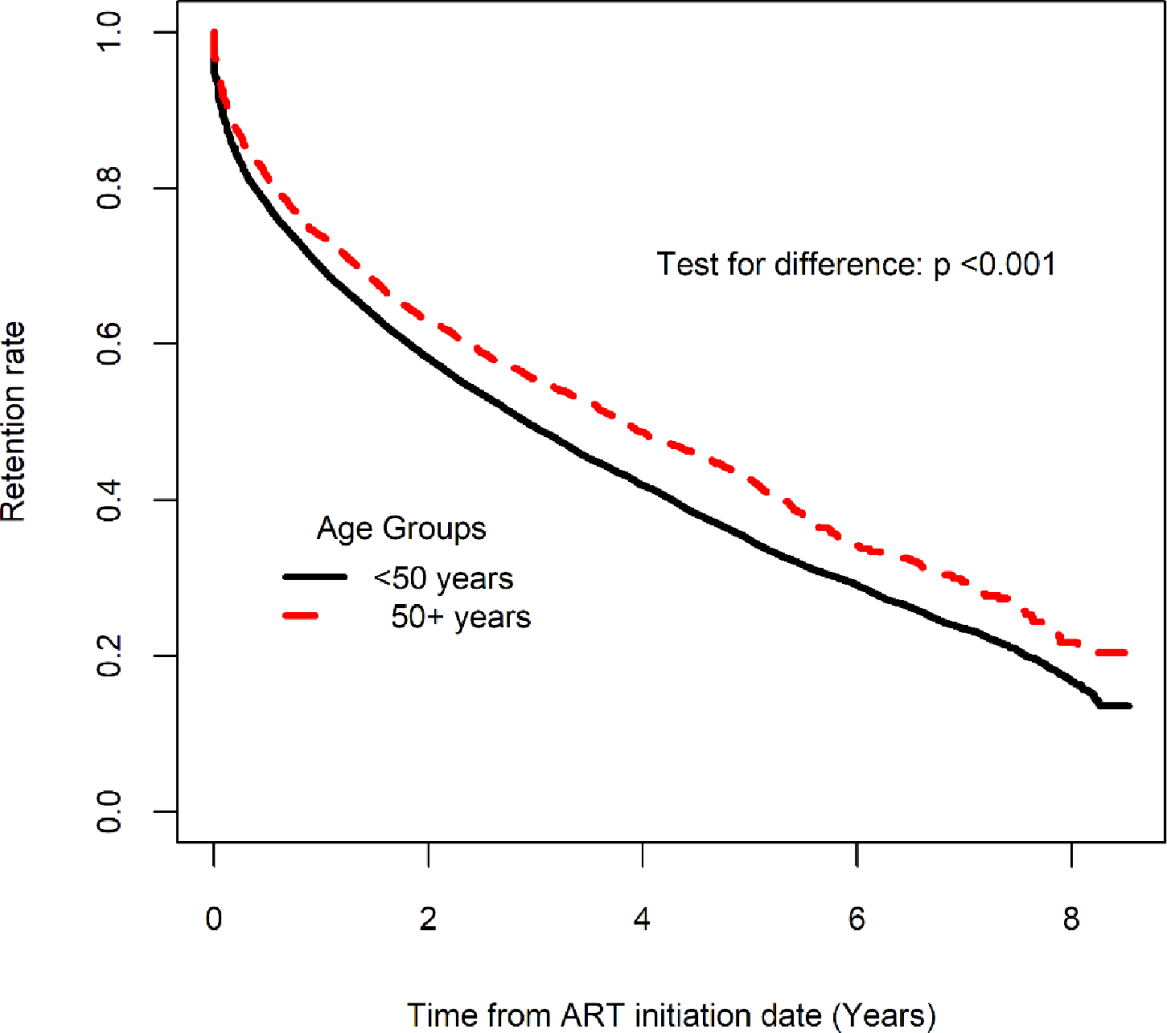
Retention rate post ART initiation among those who were initiated on ART.

The proportion of participants that were still in care at the close of the database was 44.3% among those aged ≥ 50 years, and 35.7% among those aged < 50 years. The probability of retention in care at 12, 24 and 36 months post ART initiation was 73.9% (95% CL: 72.2, 75.5), 62.9% (95% CL: 61.0, 64.7) and 55.4% (95% CL: 53.5, 57.3) among those aged ≥ 50 years compared to 69.8% (95% CL: 69.1, 70.4), 58.1% (95% CL: 57.4, 58.8) and 49.3% (95% CL: 48.6, 50.0) among < 50 years. The differences between the two groups at 12, 24 and 36 months was statistically significant, p <0.001. Participants aged ≥ 50 years had a significantly higher retention rate compared to those aged below 50 years over the entire follow up time (Log rank test, p < 0.001) “Fig 2”.

### Mortality

The older adults were more likely to die compared to the younger adults, Unadjusted HR (UHR): 1.58 (1.42, 1.76), p <0.001. The adjusted estimates showed that older adults have 35% increased risk of death compared to the younger adults, Adjusted HR (AHR): 1.35 (95% CL: 1.19, 1.52). Male participants had increased risk of death compared to female participants, AHR: 1.90 (95% CL: 1.72, 2.09). Participants who were using prophylaxis to treat opportunistic infections at enrollment, AHR: 1.22 (95% CL: 1.11, 1.34), those with advanced HIV AHR: 3.07 (95% CL: 2.72, 3.45), those who got hospitalized while on follow-up AHR: 1.39 (95% CL: 1.20, 1.60), and those who disclosed their HIV status at enrollment AHR: 1.25 (95% CL: 1.12, 1.40) had increased risk of death. “Table 4”.

**Table 4:** Predictors of retention and mortality post ART initiation.

		Mortality		Loss to Follow-up or Dead	
^†^Variable	n	Unadjusted HR (95% CL)	^ħ^Adjusted HR (95% CL)	Unadjusted HR (95% CL)	^ħ^Adjusted HR (95% CL)
Aged ≥50 vs. <50	22582	1.58 (1.42, 1.76)	1.35 (1.19, 1.52)	0.83 (0.78, 0.87)	0.83 (0.78, 0.87)
Male	22582	2.09 (1.92, 1.28)	1.90 (1.72, 2.09)	1.08 (1.03, 1.12)	1.08 (1.03, 1.12)
Urban clinic location	22582	0.82 (0.75, 0.90)	0.74 (0.68, 0.82)	0.99 (0.95, 1.03)	0.99 (0.95, 1.03)
Married	22454	0.82 (0.75, 0.89)	0.74 (0.67, 0.81)	0.85 (0.82, 0.88)	0.85 (0.82, 0.88)
^§^Using OI prophylaxis	21025	1.36 (1.24, 1.49)	1.22 (1.11, 1.34)	1.34 (1.29, 1.39)	1.34 (1.29, 1.39)
Advanced HIV status	21873	3.37 (1.04, 3.74)	3.07 (2.74, 3.45)	1.08 (1.05, 1.13)	1.08 (1.05, 1.13)
^₮^Hospitalized	21937	1.43 (1.25, 1.65)	1.39 (1.20, 1.60)	0.80 (0.75, 0.86)	0.80 (0.75, 0.86)
^§^Disclosed HIV status	22386	1.28 (1.16, 1.42)	1.25 (1.12, 1.40)	0.98 (0.94, 1.02)	0.98 (0.94, 1.02)
Sample size			19708		19708

^†^Variables measured at enrollment, Advanced HIV status = CD4 < 200 cells / mm^3^ or WHO clinical stage 3 or 4.

^₮^Hospitalized during follow-up.

^§^At baseline.

^ħ^Weighted for missing data on CD4, WHO clinical stage, and BMI, HR–Hazard Ratio, 95% CL: 95% Confidence Limits.

## Discussion

This study, conducted in the largest HIV care program in western Kenya, showed that older adults initiated on treatment were better retained in care when compared to their younger counterparts but were more likely to die due to their late presentation in care. Additionally, the proportions of older adults presenting to care at the age of 50 years and above were similar to estimates of the study conducted in four countries [27] and in another nine countries [28] in sub-Saharan African (SSA) indicating continuing increase in the numbers of older adults living with HIV. Retention in care among the older HIV-infected persons is therefore critical to their clinical outcomes and HIV prevention success.

We observed significantly higher proportions of males enrolled into care at the age of ≥50 years. Additionally, we observe that the proportion of males initiated on ART at the age of ≥50 years are significantly higher than those aged <50 years. We presume just as previous studies [29] have, that males from our population are waiting until they are symptomatic before presenting for HIV care. Our results are consistent with findings from other studies [4, 27, 28, 30, 31] that have described HIV-infected older adults in developed and developing countries. In most African settings, there exists a cultural belief that men are meant to be strong and being sick is a sign of weakness [32]. It is not until men are feeling ill and often in need of critical care that they seek care in a hospital. This is supported by our results that showed that most males were diagnosed at a hospital or healthcare setting, likely to have been seeking other unrelated care at the time of diagnosis and less likely to utilize the voluntary counselling and testing (VCT) and mobile VCT (mVCT) services available for HIV testing.

Changes in ART initiation guidelines were implemented during the study period. Initiation of ART was changed from CD4 cell counts of < 200 cells/mm^3^ to ≤ 350 cell/mm^3^ during the study period[19]. Regardless of these changes, our study showed that despite older and younger adults initiating ART at the same rate, a higher proportion of older adults were initiated on ART. These results are consistent with previous studies [27, 28, 33] that have looked at ART and outcomes among older adults in various countries in SSA. As shown in these studies, older adults in our study presented to care late into their HIV infection hence the high proportion initiated on ART. Other factors associated with ART initiation were; being married, which may mean emotional support from spouse and a sense of responsibility for their health and those of their loved ones and being diagnosed with an opportunistic condition, which could be linked to advanced HIV infection.

Retention in care among HIV-infected persons is critical to HIV treatment and prevention success. We observed better retention at 12, 24 and 36 months post ART initiation among older adults compared to younger adults. Our findings compare to other studies conducted in African setting [18, 27, 33] that have shown that despite slower cognitive function and increased co-morbidities, older adults were more likely to be engaged in care for longer than their younger counterparts. We found other factors associated with increased retention that included; having disclosed HIV status and being married. These factors have also been documented by other studies [9, 11, 33], conducted in SSA that looked at factors associated with LTFU among older adults. Our study findings, however, differ with that by Yehia et al. [34] that looked at the impact of age in retention to care and showed that younger adults were more likely to be retained in care and attain viral suppression. Yehia’s study was conducted in a resource-rich setting which differ in many ways with our resource limited settings. High retention among older adults could also indicate better viral suppression [7] among the group.

Mortality rates among older adults were significantly higher than those in younger adults. This study showed that 24% of older adults died within the first years of initiating ART. These findings are similar to those from other sub-Saharan African countries that have demonstrated increased mortality after ART initiation among older adults [9, 29, 35–37]. Higher mortality rates among older adults in this study may be explained by the late presentation in care with low CD4 counts, and advanced disease stage (WHO stage III and IV) characterized by increased opportunistic diseases. In addition, since causes of death were inconsistently collected in our setting, we hypothesize that ART toxicities and comorbidities likely to occur among older adults would have contributed to the high mortality. There is need to look at how comorbidities and ART toxicities in our population contributed to increased mortality among the older adults.

To the best of our knowledge, this study presents data from the largest single program on retention of older adults on ART. Other studies that have presented data on retention have focused on specific phase in HIV care continuum or have largely described adults in general. We believe this study present unique characteristics that are useful for programs providing care to HIV infected person and want to target older adults.

There were limitations in our study that need to be considered. Our study utilized routinely collected clinical data, often characterized by missing or incomplete data. In our setting, routine measurement of CD4 counts were not done for every patient. We therefore, had patients missing significant proportion of their data on CD4 counts, WHO clinical staging, and weight and height, important factors for BMI calculation. As described in our analysis methods, we used inverse probability weighting (IPW) to account for these missing covariates. Our approach resulted in similar survival distributions among those with complete covariate data and those with partially complete covariate data. Also, given that old age and other co-morbidities increase the risk of death among older adults with HIV, it is highly likely that the high mortality seen among the older adults could be as a result of other age-related conditions besides HIV.

## Conclusion

This study found that older adults have better retention in care at 12, 24 and 36 months post ART initiation compared to younger adults. However, older adults were more likely to die perhaps due to the late presentation in care. Efforts to actively find older adults early in the HIV infection and initiate them on ART is important in enhancing their health outcomes.

## Supporting information

S1 File Retention in care among older adults–western Kenya dataset. (XLS)
